# Modeling of mesenchymal hybrid epithelial state and phenotypic transitions in EMT and MET processes of cancer cells

**DOI:** 10.1038/s41598-018-32737-z

**Published:** 2018-09-25

**Authors:** Peng He, Kang Qiu, Ya Jia

**Affiliations:** 1 Department of Physics and Institute of Biophysics, Central China Normal University, Wuhan, 430079 China; 2 Department of Mathematics and Physics, Xuzhou Medical University, Xuzhou, 221004 China

## Abstract

Based on the transcriptional regulatory mechanisms between microRNA-200 and transcription factor ZEB in an individual cancer cell, a minimal dynamic model is proposed to study the epithelial-mesenchymal transition (EMT) and mesenchymal-epithelial transition (MET) processes of cancer cells. It is shown that each cancer cell can exit in any of three phenotypic states: the epithelial (E) state, the mesenchymal (M) state, and the epithelial/mesenchymal (E/M) hybrid state, and the state of cancer cell can interconvert between different states. The phase diagram shows that there are monostable, bistable, and tristable phenotypic states regions in a parameters plane. It is found that different pathway in the phase diagram can correspond to the EMT or the MET process of cancer cells, and there are two possible EMT processes. It is important that the experimental phenomenon of E/M hybrid state appearing in the EMT process but rather in the MET process can be understood through different pathways in the phase diagram. Our numerical simulations show that the effects of noise are opposite to these of time delay on the expression of transcription factor ZEB, and there is competition between noise and time delay in phenotypic transitions process of cancer cells.

## Introduction

Modeling of microscopic regulatory mechanisms in single cancer cell is a crucial step towards understanding macroscopic physiological phenomena of cancer cells. The epithelial-mesenchymal transition (EMT) and its reverse process (mesenchymal-epithelial transition, MET) play a critical role in embryonic development, tissue regeneration, and cancer metastasis^1,2^.

It is well known that most cancer patients die from their disease as a result of metastasis^3^. Cancer cells leave the primary tumor and seed new secondary tumors in distant organs through blood circulation and lymphatic vessels, where the EMT and MET processes promote cancers metastasis and make cancers aberrantly activated. During the metastasis, cancer cells undergoing EMT lose their cell-cell adhesion gradually, and gain migratory capacities. Then, the cancer cells invade into the basement membrane by entering the blood vessels as circulating tumor cells (CTCs)^4^. The CTCs undergoing the reversal process MET settle down, and form new secondary tumors when the CTCs arrive at distant organs^5^.

In the processes of EMT and MET, cancer cells can exit three phenotypic states: the epithelial (E), the mesenchymal (M), and the epithelial/mesenchymal (E/M) hybrid state, and the phenotype of cancer cells interconvert between three states. The E/M hybrid state is a combination of epithelial and mesenchymal traits which could promote cancer cells to move collectively during mammary gland formation, trachea development, and wound healing^6,7^. In previous investigations, the E/M hybrid state of cancer cells was considered as a metastable phenotypic state by many researchers, that is, the hybrid phenotypic state is a transient state during EMT process^8,9^. However, it was found that some transcription factors such as OVOL and GRLH2 (the phenotypic stability factors) can make the hybrid phenotype to be a steady state^10^. Recent experiments demonstrated that the E/M hybrid phenotypic state had been observed in the EMT process, whereas it was not found in the reverse MET process^11,12^.

**Figure 1:**
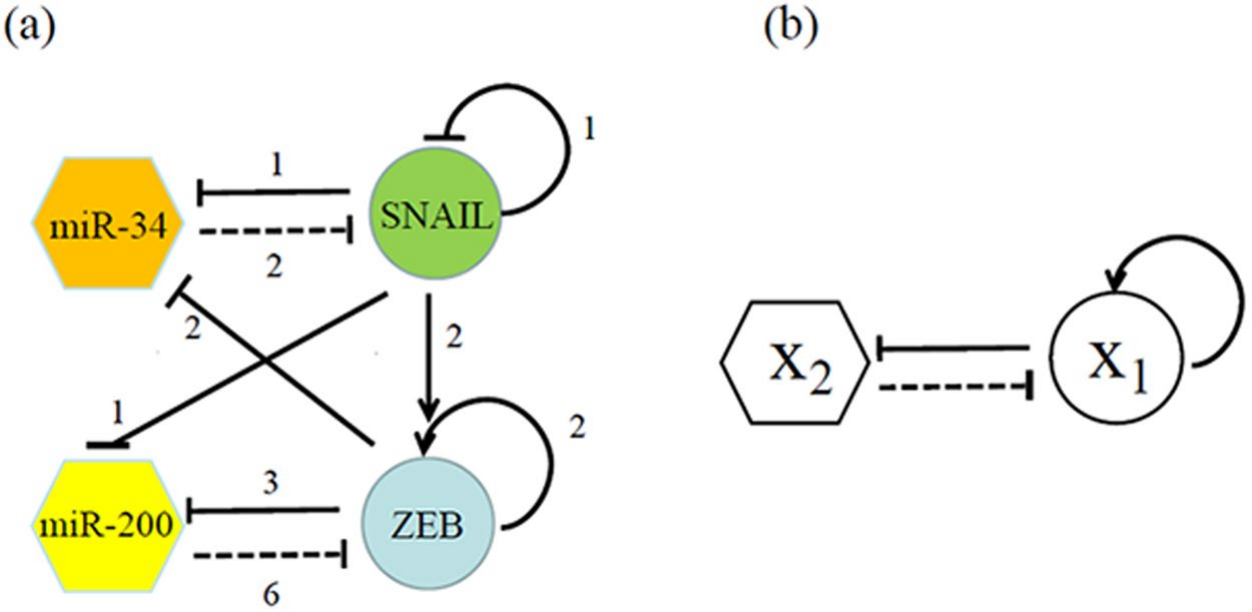
(**a**) The core regulatory network^19^ is composed of four components (two families of E-box binding transcription factors SNAIL and ZEB, and two families of microRNAs miR-34 and miR-200). (**b**) A general minimal dynamic model of the core regulatory network.

It is believed that the multi-phenotypic states of cancer cells arise in a cancer cell with different genes expression levels. It was shown that different phenotype of cancer cells is induced by various internal and external signals such as HIF-1α, p53, TGF-β, HGF, FGF, EGF, Notch, Wnt, and Hedgehog^2,13–18^. Recently, in order to understand the existence of E/M hybrid phenotypic state of cancer cell, a core regulatory network governing epithelial-mesenchymal plasticity had been proposed by Lu *et al*.^19^, the core regulatory network is composed of four components (two families of E-box binding transcription factors SNAIL and ZEB, and two families of microRNAs (miRs) miR-34 and miR-200). There are two double negative feedback loop between TFs and miRs in the regulatory network, that is, the miR-34/SNAIL and the miR-200/ZEB mutually inhibiting loops as shown in Fig. 1(a), and it was found that the miR-200/ZEB module functions as a ternary switch, allowing not only for the epithelial and mesenchymal phenotypes but also for a hybrid phenotype^10,19^.

On the one hand, a large number of experiments confirmed that non-coding RNAs can regulate EMT, in particular, the expression of miR-200 family is strongly associated with epithelial differentiation, and a reciprocal feedback loop between the miR-200 family and the ZEB family of transcription factors tightly controls both EMT and MET^20–22^. Moreover, the EMT and MET mediated by miR-200/ZEB module was found in human various tumor cells, for example, in the colon cancer cells^23,24^, in the breast cancer cells^25,26^, etc. Although there are two double negative feedback loops between TFs and miRs in the core regulatory network^19^, that is, the miR-34/SNAIL and the miR-200/ZEB mutually inhibiting loops as shown in Fig. 1(a) with four variables, it was also showed that the miR-34/SNAIL module is a monostable circuit functions as a noise-buffering integrator, and the miR-200/ZEB module functions as a ternary switch (i.e., miR-200/ZEB acts as the decision making module for cancer cells to undergo partial or complete EMT), allowing not only for the epithelial and mesenchymal phenotypes but also for a hybrid phenotype, and these theory-based findings are consistent with several experimental findings^10,19^. Therefore, a minimal dynamic model of miR-200/ZEB module could be sufficient and appropriate to simulate EMT/MET.

On the other hand, both fluctuation and time delay are ubiquitous in various intracellular signal networks^27–30^, where the noise could originate from the random births and deaths of individual molecules or the fluctuations in biochemical reactions, and the time delay could arise from finite propagation speeds of signal molecules. The effects of both noise and time delay have been widely studied in various biological systems, such as the genetic regulatory networks^31,32^, the neuronal systems^33–36^, etc.

Interesting questions now arise: is there a minimal dynamic model to understand the E/M hybrid state in the EMT process? What are the effects of noise and time delay on the minimal regulatory mechanism of multiple phenotypic states for the cancer cell?

In this paper, based on the core regulatory network proposed by Lu *et al*.^19^, a general minimal dynamic model with two variables is proposed to understand the EMT and MET processes and the effects of both noise and time delay. By utilizing the general minimal mathematical model, it can be found that each cancer cell may exit in any of three phenotypic states (E, M, and E/M hybrid states), there are two possible EMT pathways, and the phenomenon of E/M hybrid state in the EMT processes but rather in the MET process observed by experiments^11,12^ can be revealed through different pathways in a parameters plane (phase diagram). In addition, the effects of noise and time delay on the interconversions between phenotypic states are discussed by using numerical simulations.

## Model and Method

The four components model of the miR-34/SNAIL and the miR-200/ZEB mutually inhibiting loops^19,37^ of cancer cells is shown in Fig. 1(a), it can be found that the interactions between transcription factors and microRNAs have symmetrical properties. The expression of SNAIL from the miR-34/SNAIL module can been considered as a external signal action on the miR-200/ZEB module, on the contrary, the expression of ZEB from the miR-200/ZEB module can be considered as a external signal action on the miR-34/SNAIL module. Therefore, one can simplify core regulatory network by a general minimal regulatory model with two variables as shown by Fig. 1(b), in this paper, *X*_1_ represents the transcription factor ZEB family, and *X*_2_ represents the miR-200, and the SNAIL signal activates the expression of ZEB and inhibits the expression of miR-200 simultaneously.

In the deterministic description, the dynamic model of the minimal regulatory motif Fig. 1(b) can be described by following ordinary differential equations in dimensionless:

1 $$\frac{d{x}_{1}}{dt}=\frac{a{x}_{1}^{{n}_{1}}}{{\theta }^{{n}_{1}}+{x}_{1}^{{n}_{1}}}+\frac{c{\theta }^{{n}_{2}}}{{\theta }^{{n}_{2}}+{x}_{2}^{{n}_{2}}}-{k}_{1}{x}_{1}={F}_{1}({x}_{1},{x}_{2}),$$

2 $$\frac{d{x}_{2}}{dt}=\frac{b{\theta }^{{n}_{3}}}{{\theta }^{{n}_{3}}+{x}_{1}^{{n}_{3}}}-{k}_{2}{x}_{2}={F}_{2}({x}_{1},{x}_{2}),$$

where *x*_1_ and *x*_2_ are the expression levels of transcription factors ZEB family and miR-200. *a* is the activation strength of ZEB induced by both SNAIL signal and itself, *b* is the inhibited strength of miR-200 induced by both SNAIL signal and *X*_1_, *c* is the inhibited strength of ZEB by miR-200, and *k*_1_ and *k*_2_ are the self-degradation rates of ZEB and miR-200, respectively. Parameters *n*_1_, *n*_2_, and *n*_3_ are the Hill coefficients which control the steepness of the sigmoidal function, and *θ* is a threshold. Here, the parameter values are *n*_1_ = 2, *n*_2_ = 6, and *n*_3_ = 3 which are on the basis of the different binding sites obtained from experiments^19^, and *k*_1_ = *k*_2_ = 1.0 and *θ* = 0.5^38^. For simplicity, the inhibited strength of ZEB by mir-200 is set as constant value *c* = 1.0. Here, it was hypothesized that the negative regulations on *X*_2_ by both SNAIL and *X*_1_ (and the positive regulations on *X*_1_ by both SNAIL and itself) can simultaneously occur, thus, the regulations of first item in the right side of Eqs (1 and 2) follow an “and” rather than “or” logic.

In the stochastic description, above general dynamic model Eqs (1, 2) are written by following Langevin equations

3 $$\frac{d{x}_{1}}{dt}={F}_{1}({x}_{1},{x}_{2})+{\xi }_{1}(t),$$

4 $$\frac{d{x}_{2}}{dt}={F}_{2}({x}_{1},{x}_{2})+{\xi }_{2}(t),$$

where ${\xi }_{1}(t)$ and ${\xi }_{2}(t)$ are the Gaussion white noises with zero mean and auto-correlation functions $\langle {\xi }_{1}(t){\xi }_{1}(s)\rangle =2{D}_{1}\delta (t-s),\langle {\xi }_{2}(t){\xi }_{2}(s)\rangle =2{D}_{2}\delta (t-s)$. We consider a homogeneous situation (i.e., *D*_1_ = *D*_2_ = *D*), which means that the noises in Eqs (3 and 4) represent the total effect of intrinsic and extrinsic noises. The probability distribution $P({x}_{1},{x}_{2},t)$ of system (3) and (4) obeys the Fokker-Planck equation^38,39^:

5 $$\begin{array}{rcl}\frac{\partial P({x}_{1},{x}_{2},t)}{\partial t} & = & -\frac{\partial }{\partial {x}_{1}}{F}_{1}({x}_{1})P({x}_{1},{x}_{2},t)-\frac{\partial }{\partial {x}_{2}}{F}_{2}({x}_{1},{x}_{2})P({x}_{1},{x}_{2},t)\\  &  & +\,D(\frac{{\partial }^{2}}{\partial {x}_{1}^{2}}+\frac{{\partial }^{2}}{\partial {x}_{2}^{2}})P({x}_{1},{x}_{2},t).\end{array}$$

The stationary probability ${P}_{st}({x}_{1},{x}_{2})$ obtained from the Fokker-Planck Eq. (5) can be used to indicate the phenotypes proportion distribution of cancer cells. In the equilibrium case, the potential function $U({x}_{1},{x}_{2})$ for non-equilibrium system can be defined by the stationary probability:

6 $$U({x}_{1},{x}_{2})=-\,\mathrm{ln}\,[{P}_{st}({x}_{{\rm{1}}},{x}_{2})]{\rm{.}}$$

Each minimum of *U*(*x*_1_; *x*_2_) corresponds to one state (or phenotype) of a cancer cell, and the phenotypic switching of cancer cells is understood that the state of the cancer cell moves from one minimum of potential landscape to another.

To quantify the properties of phenotypic switching between states in the case of multiple phenotypic states coexistence, one can calculate the escape time from one steady state of *U*(*x*_1_, *x*_2_) to another. A rigorous definition of escape time is provided by the mean first-passage time (MFPT) of a stochastic process *y*(*t*) to reach the point *y*_2_ with initial condition *y*(*t* = 0) = *y*_1_^40,41^:

7 $$\tau ={\int }_{{y}_{1}}^{{y}_{2}}\frac{dy}{D(y){P}_{st}(y)}{\int }_{\infty }^{y}{P}_{st}(q)\,dq.$$

In order to study the time delay arise from finite propagation speeds of regulatory molecule, here we assume that the activation of ZEB induced by itself through the post-transcriptional regulation has a time delay *τ*, thus, Eq. (3) is rewritten by:

8 $$\frac{d{x}_{1}}{dt}=\frac{a{[{x}_{1}(t-\tau )]}^{{n}_{1}}}{{\theta }^{{n}_{1}}+{[{x}_{1}(t-\tau )]}^{{n}_{1}}}+\frac{c{\theta }^{{n}_{2}}}{{\theta }^{{n}_{2}}+{x}_{2}^{{n}_{2}}}-{k}_{1}{x}_{1}+{\xi }_{1}(t).$$

## Results

Although the expression levels of genes are not the only factor, they are the major influencing factor to determine the phenotypes of a cancer cell. According to the expression levels of *X*_1_ and *X*_2_ in the general minimal dynamic model Eqs (1, 2), one can assume that 0 represents the relatively low expression level, 1 represents the relatively high expression level, and 1/2 represents the relatively intermediate expression level. There are three states (*x*_1_, *x*_2_), which are the (1, 0), (0, 1), and (1/2, 1/2) states. In the EMT and MET processes, the phenotypic states of cancer cells arise in a cancer cell with different genes expression levels^10,19,42^. Thus, the (1, 0) state obtained from the general minimal dynamic model means the high miR-200 and low ZEB expression level which corresponds to the epithelial phenotype (E) of cancer cell, the (0, 1) state means the low miR-200 and high ZEB expression level which corresponds the mesenchymal phenotype (M) of cancer cell, and the (1/2, 1/2) state means the intermediate expression level of both miR-200 and ZEB which corresponds to the epithelial/mesenchymal hybrid phenotype of cancer cell.

### Phase diagram in (*a*, *b*) parameters plane

**Figure 2:**
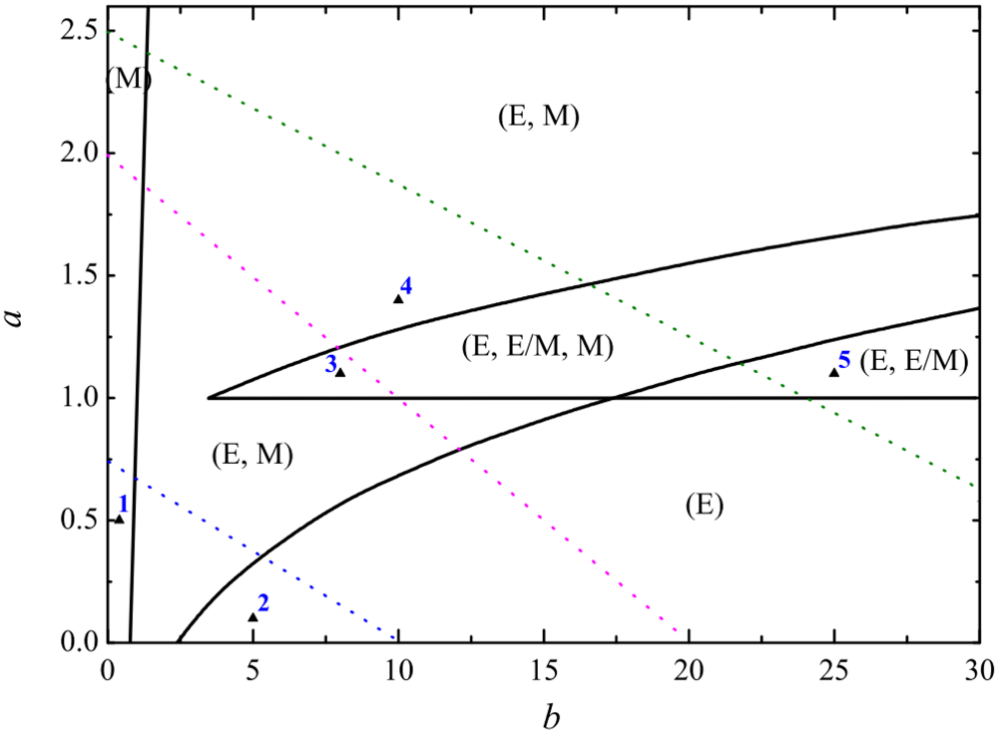
The phase diagram of deterministic model in the (*a*, *b*) parameters plane. The five regions in the phase plane correspond to (M), (E), (E, M), (E, E/M), and (E, E/M, M) states of cancer cell, respectively. The pink dotted line corresponds to one possible EMT process, and the green dotted line corresponds to the other possible EMT process with the increasing of parameter *a*. The blue dotted line corresponds to a possible MET process with the decreasing of parameter *a*.

A steady state of the dynamic model corresponds to a phenotypic state of cancer cell. By using our minimal dynamic model, a phase diagram of phenotypic states of single cancer cell is drawn in parameters (*a*, *b*) plane as shown by Fig. 2. The phase diagram shows that there are five regions: two monostable regions which correspond to the E-(1, 0) sate or the M-(0, 1) state, two bistable regions which correspond to the coexistence of E-(1, 0) and M-(0, 1) states or the coexistence of E-(1, 0) and E/M hybrid-(1/2, 1/2)) hybrid states, and a tristable region which corresponds to the coexistence of E-(1, 0), E/M hybrid-(1/2, 1/2), and M-(0, 1) states.

**Figure 3:**
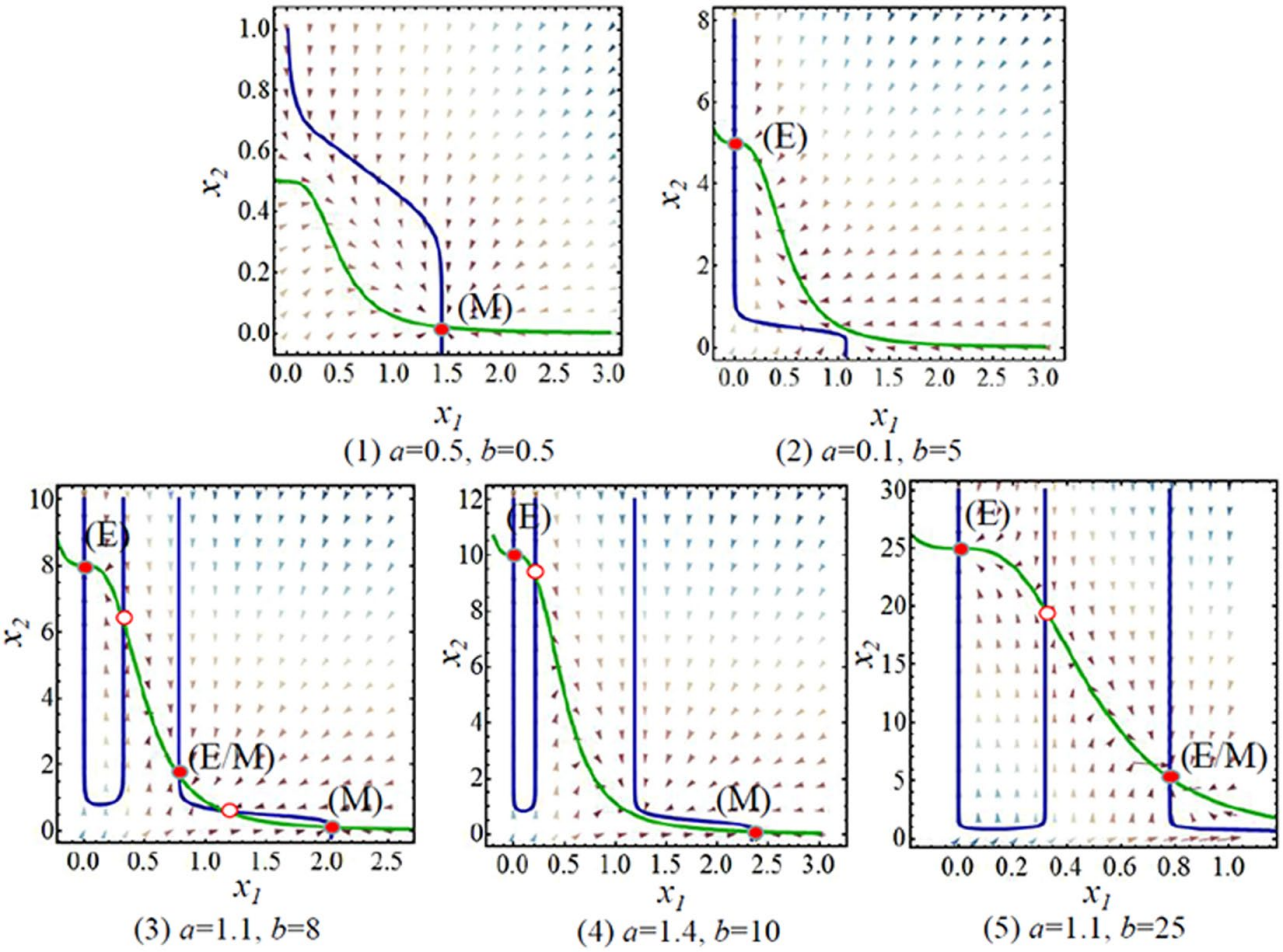
The flow pattern map corresponds to the five black triangle points (1, 2, 3, 4, and 5) in Fig. 2. The blue and green lines are the nullclines, the solid red points denote the steady states (the cell phenotypes), and the circle red points denote the unsteady states.

One point in each regions (see the black triangle points in Fig. 2) was chosen to analyse the stability of deterministic trajectories of dynamic system. Figure 3 shows there is one stability point which correspond to the single E state (Fig. 3(1)) and the single M state (Fig. 3(2)) in the monostable regions. There are two stability points which correspond to the coexistence of (E, M) states (Fig. 3(4)), and the coexistence of (E, E/M) states (Fig. 3(5)) in the bistable region. There are three stability points which correspond the coexistence of (E, E/M, M) states (Fig. 3(3)) in the tristable region.

The coexistence of multiple phenotypic states and the phenotypes transitions of cancer cells had been confirmed by some experiments in the EMT process^43–45^. It can be found from the phase diagram Fig. 2, that the epithelial (E) phenotype is appeared in all multiple phenotypic states regions, which implies that epithelial state is a fundamental phenotypic state and more robust than other states^46,47^.

### EMT and MET processes of cancer cells

It was demonstrated by experiments^11,12^ that the E/M hybrid phenotypic state of cancer cells can be observed in the EMT process, but can not be observed in the reverse MET process. On the one hand, from the core regulatory network (see Fig. 1(a)) governing epithelial-mesenchymal plasticity^19^, it can be found that the SNAIL signal activates the expression of ZEB and inhibits the expression of miR-200 simultaneously. On the other hand, there are two key parameters in our general minimal dynamic model, one is the activation strength *a* of ZEB which can be induced by both SNAIL signal and itself, and the other is the inhibited strength *b* of miR-200 which can be induced by both SNAIL signal and ZEB. That is, the actions of SNAIL signal on the miR-200/ZEB mutually inhibiting loop can cause the variations of parameters *a* and *b*. Thus, under the condition of limited SNAIL molecules, the SNAIL signal results in the increasing of *a* and the decreasing of *b*. For simplicity, here we assume that *b* is a linear function of *a* due to the SNAIL signal, such as *b* = −*αa* + *β*, where *α* and *β* are positive constants, *a* and *b* ≥ 0.

#### The EMT process

**Figure 4:**
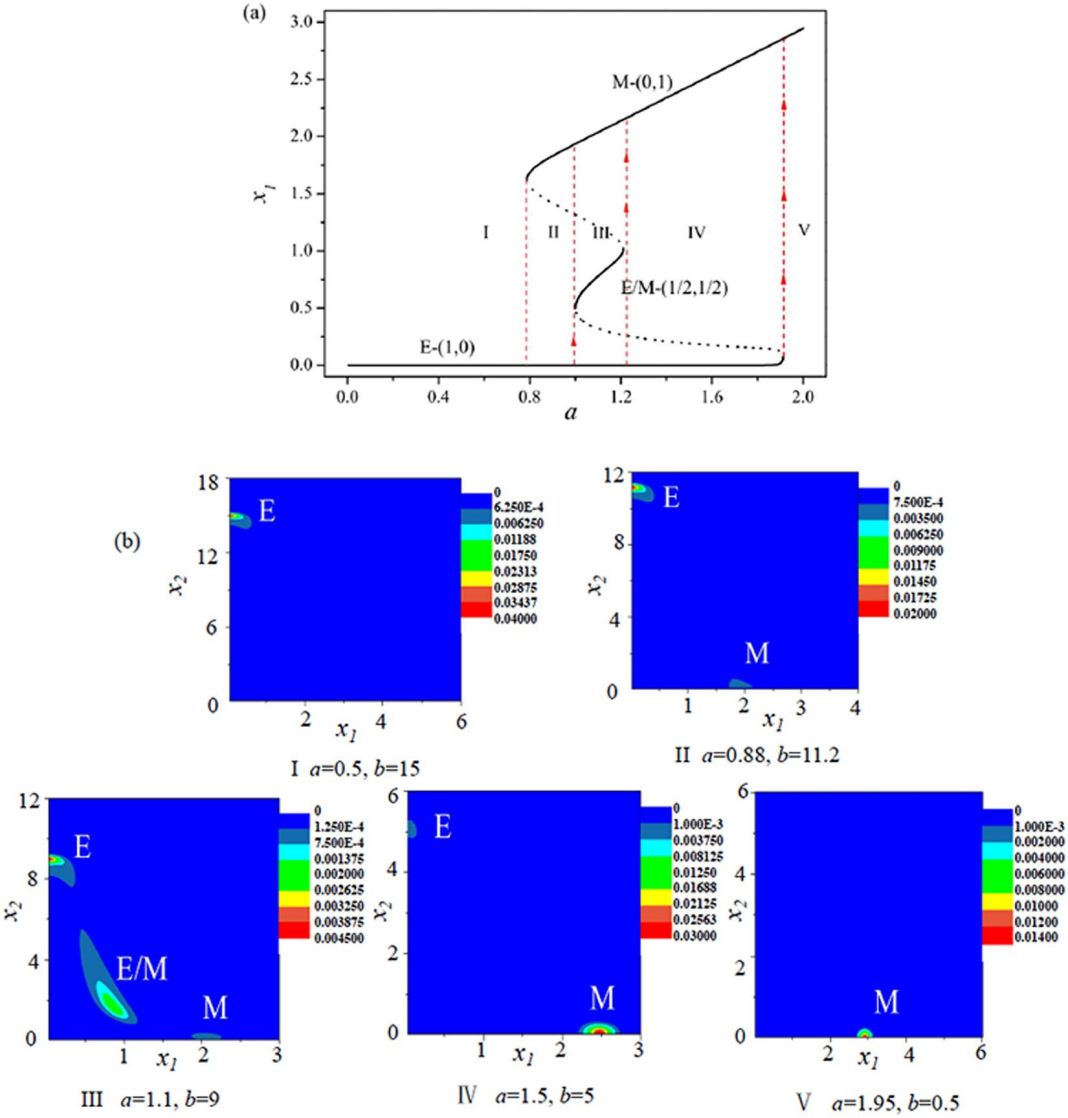
The E/M hybrid state can be observed in a possible EMT process (the pink dotted line with the increasing of *a* in Fig. 2). (**a**) The bifurcation diagram of *x*_1_ with the increasing of *a*. (**b**) Stationary probability distributions *P*_*st*_(*x*_1_, *x*_2_) correspond to the five regions (I, II, III, IV, and V) in (**a**). *D* = 0.01.

In order to illustrate the EMT process, for example, the pink dotted line (with *b* = −10*a* + 20) in Fig. 2 represents a possible pathway that a cancer cell undergoes a EMT process with the increasing of activation strength *a* of ZEB. Figure 4(a) shows that, with the increasing of activation strength *a* of ZEB which can be induced by external signal (such as the SNAIL), the cancer cell undergoes a succession of transitions: the single E phenotypic state → the coexistence of (E, M) states → the coexistence of (E, E/M, M) states → the coexistence of (E, M) states → the single M phenotypic state.

Figure 4(b) shows the stationary probability *P*_*st*_(*x*_1_, *x*_2_) at the three phenotypic states of cancer cell in different region of Fig. 4(a). With the variations of parameters *a* and *b*, although the cancer cell experiences twice coexistence of (E, M) states in the EMT possess, the probability of each state is very different by comparing Fig. 4b(II) with Fig. 4b(IV).

**Figure 5:**
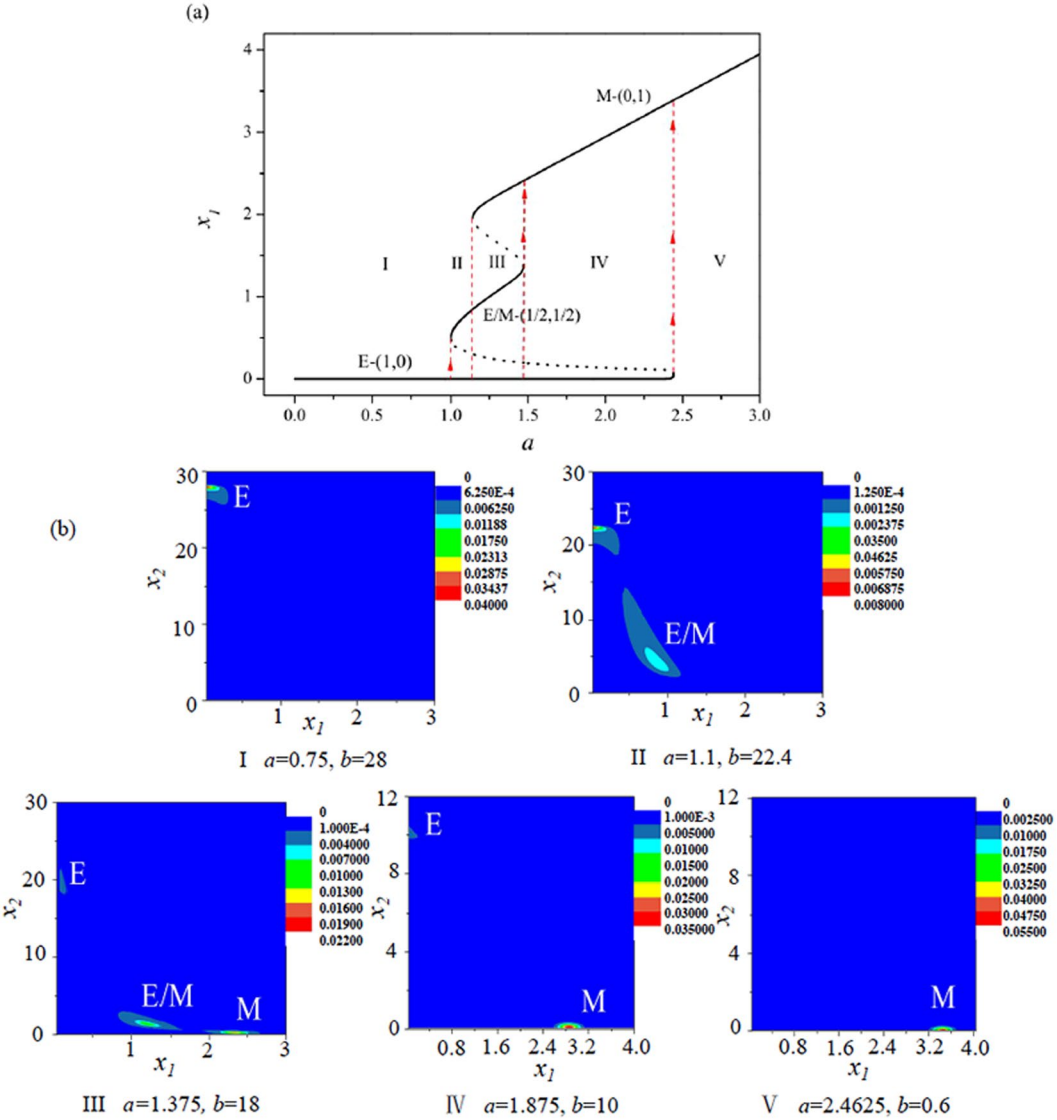
The E/M hybrid state can be observed in another possible EMT process (the green dotted line with the increasing of *a* in Fig. 2). (**a**) The bifurcation diagram of *x*_1_ with the increasing of *b*. (**b**) Stationary probability distributions *P*_*st*_(*x*_1_, *x*_2_) correspond to the five regions (I, II, III, IV, and V) in (**a**). *D* = 0.01.

It is very important that there is another possible EMT process as shown by the green dotted line (with *b* = −16*a* + 40) in Fig. 2. Figure 5(a,b) show that, with the increasing of activation strength *a* of ZEB, the cancer cell undergoes a succession of transitions: E → (E, E/M) → (E, E/M, M) → (E, M) → M. The difference between above two EMT paths is the second phase: one is the coexistence of (E, M) states, and the other is the coexistence of (E, E/M) states. However, in both EMT processes, it is interesting that the E/M hybrid phenotypic state can be observed in the EMT processes. It is also found that the E state always exist until the the activation strength *a* of ZEB is too large, which suggests that the E state may be a fundamental phenotype for the cancer cell which is consistent with the experimental observations^46,47^.

#### The MET process

To illustrate the MET process, we considered the blue dotted line (with *b* = −13.33*a* + 10) in Fig. 2, which represents a possible pathway that a cancer cell undergoes a MET process with the decreasing of activation strength *a* of ZEB.

**Figure 6:**
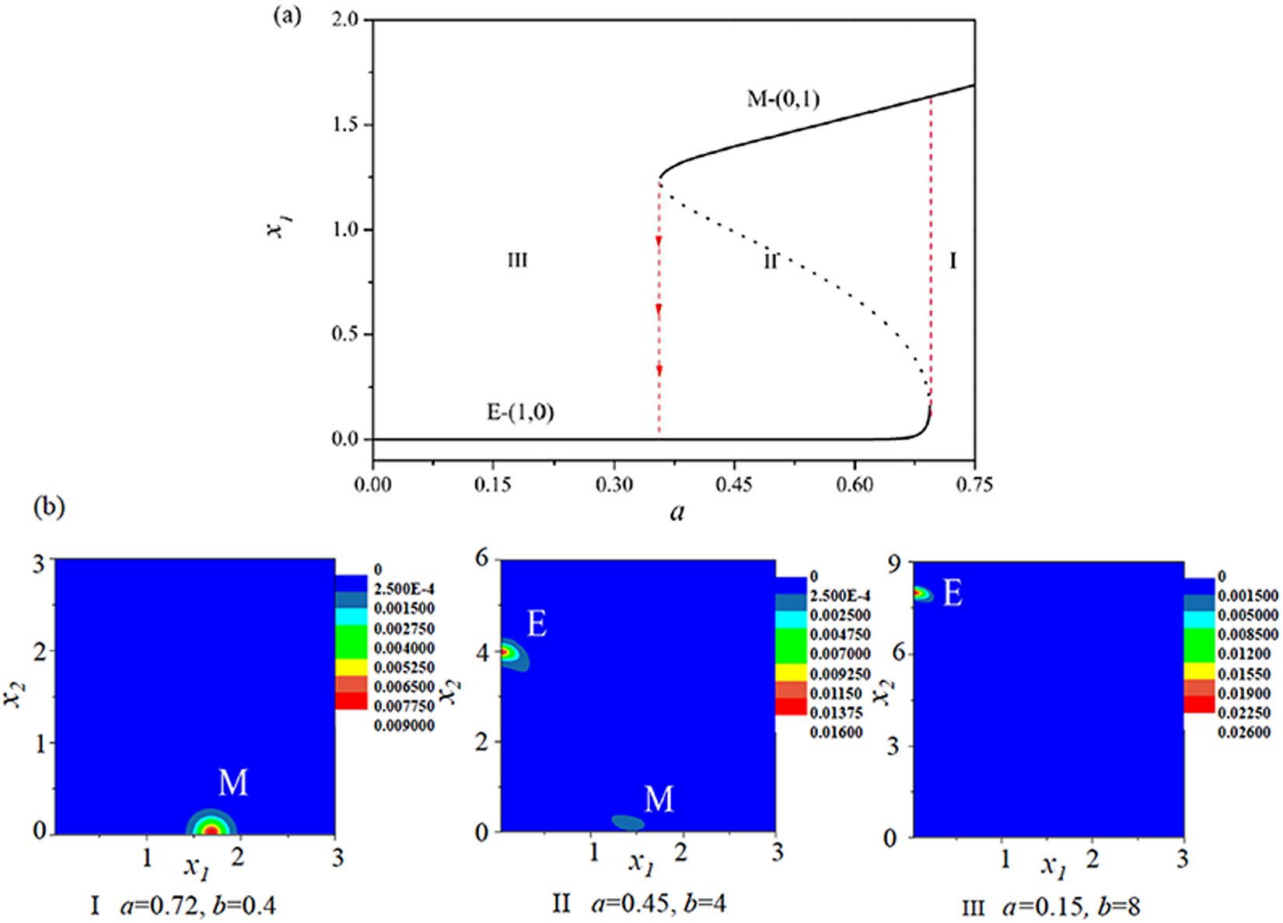
The E/M hybrid state can not be observed in the MET process (the blue dotted line with the decreasing of *a* in Fig. 2). (**a**) The bifurcation diagram of *x*_1_ with the decreasing of *a*. (**b**) Stationary probability distributions *P*_*st*_(*x*_1_, *x*_2_) correspond to the three regions (I, II, and III) in (**a**). *D* = 0.01.

Figure 6(a) shows that, with the decreasing of activation strength *a* of ZEB, the cancer cell will undergo a succession of transitions: M → (E, M) → E. Figure 6(b) shows the stationary probability *P*_*st*_(*x*_1_, *x*_2_) at the two phenotypic states of cancer cell in different region of Fig. 6(a). It can be seen that the probability of M state is decreased, however, the probability of E state is increased with the variations of parameters *a* and *b*. In above MET process, it is interesting that the cancer cell can not exist the E/M hybrid phenotypic state, that is, there is no the E/M hybrid state in the MET process.

By utilizing our general minimal mathematical model with two variables, the different phenotypic states of cancer cell as shown in ref.^19^ can be reproduced. Most importantly, the phenomenon of E/M hybrid state in the EMT processes but rather in the MET process observed by experiments^11,12^ can be understood through different pathways in the parameters (*a*, *b*) plane.

### Phenotypic transitions in tristable region

The regulation of cancer cell state decisions is of particular significance in tumor pathobiology. The cancer cells frequently exist in any of possible phenotypic states, and cancer cells can interconvert between different states. There are extensive studies^2–4^ on the phenotypes E and M of cancer cells. However, the E/M hybrid state of cancer cells is more complex and also important in physiology and pathology^10^ since the hybrid phenotype with the mixed traits can lead to cancer cells collective migration. The hybrid phenotype can induce the cancer cells maximum cellular plasticity^6^.

In order to discuss the phenotypes transitions in the tristable region where a cancer cell can be found in the multiple states (E, E/M, M) coexistence. Here, a point (*a*, *b*) = (1.1, 8) in the tristable region was chosen to study the phenotypes transitions between different states under different noise intensity.

**Figure 7:**
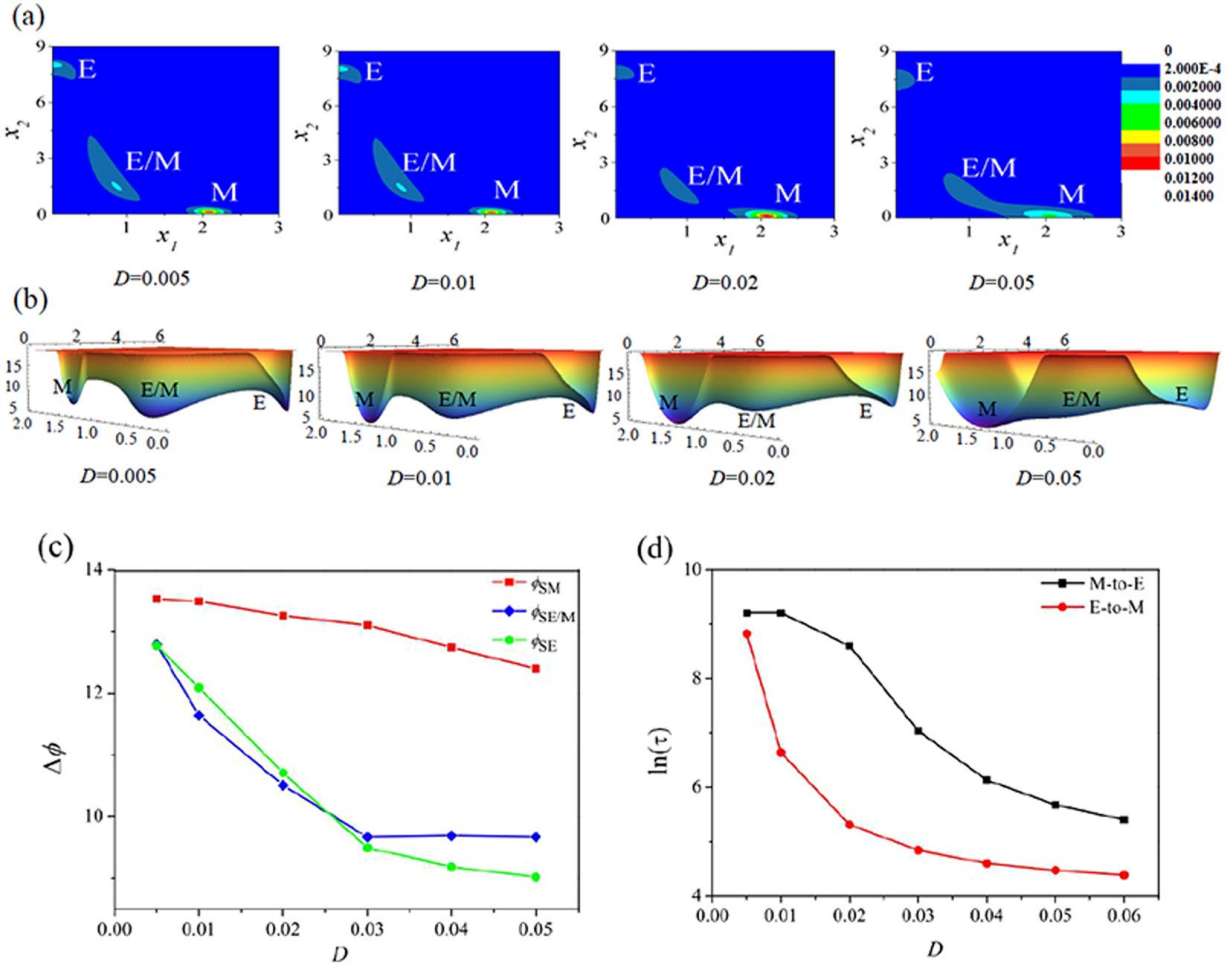
Phenotypes transitions induced by noise at point (*a* = 1.1, *b* = 8) in the tristable region of Fig. 2 (**a**,**b**) are the stationary probability *P*_*st*_(*x*_1_, *x*_2_) and the potential function *U*(*x*_1_, *x*_2_) with different noise intensity, respectively. (**c**,**d**) Are the height of barriers and the mean first passage times between two attractors with the increase of noise intensity.

With the increasing of noise intensity, Fig. 7(a) shows that the stationary probability of M state is gradually increased, and the potential landscapes corresponding to Fig. 7(a) are shown in Fig. 7(b). It is found that the cancer cell has a large probability of transition into the M state through the E/M hybrid phenotype. When the cancer cell is in the M state, it becomes more migratory and invasive^4,6^. To quantify the properties of phenotypic switching between different states, one can calculate the mean first passage time (MFPT) from one steady state to another according to Eq (7). In addition, the barrier height of minima of potential function Eq (6) can also be used to imply the properties of phenotypic switching, the height of barriers of two attractors (e.g., the *A* and *B* states) is defined by: *φ*_*SA*_ = *φ*_*S*_ − *φ*_*A*_ and *φ*_*SB*_ = *φ*_*S*_ − *φ*_*B*_^38^, where *S* is the saddle point between *A* and *B* states.

With the increase of the noise intensity, Fig. 7(c) shows that the barrier heights of E, E/M hybrid, and M states are decreased, which means the larger noise intensity can lead to the steady states more instability, and the noise lets cancer cell easily escapes from the steady states. Figure 7(d) shows the MFPT from E state to M state or from M state to E state, respectively. Although all MFPTs are decreased with the increasing of noise intensity, the MFPT from E to M state is smaller than that from M to E state, which means that the M phenotype is much more stable than the E phenotype for cancer cell.

Interestingly, with the increase of noise intensity, it is found that the barrier height *φ*_*SE/M*_ of E/M hybrid phenotype (see the blue line in Fig. 7(c)) is decreased, and then reaches a platform value when the noise intensity is larger than a critical value *D*_*c*_ = 0.03, that is, the E/M hybrid state always exists when *D* > *D*_*c*_. The cancer cell in the E/M hybrid state can acquire the capabilities of resistance and robustness to against the high pressure environment. It was reported that cancer cells in the E/M hybrid phenotype exhibit stemness characters during EMT in developmental, regenerative, as well as pathological contexts^48–51^, and related to drug resistance^52–54^.

### Effects of time delay and noise on phenotypic transitions

The multi-phenotypic states of cancer cells arise in a cancer cell with different genes expression levels, for example, the low ZEB expression corresponds to E phenotype, the high ZEB expression corresponds to M phenotype, and the intermediate ZEB expression corresponds to E/M hybrid phenotype^19^. In order to discuss the effects of time delay and noise on the phenotypic states transitions, two points in the phase diagram Fig. 2 were chosen as examples: one is the point (*a*, *b*) = (0.1, 1) which is in a bistable region, and the other is the point (*a*, *b*) = (1.1, 8) which is in the tristable region. The effects of time delay and noise on the expression level *x*_1_ of ZEB are discussed by using Eqs (3 and 8) through numerical simulations.

#### In the bistable region

**Figure 8:**
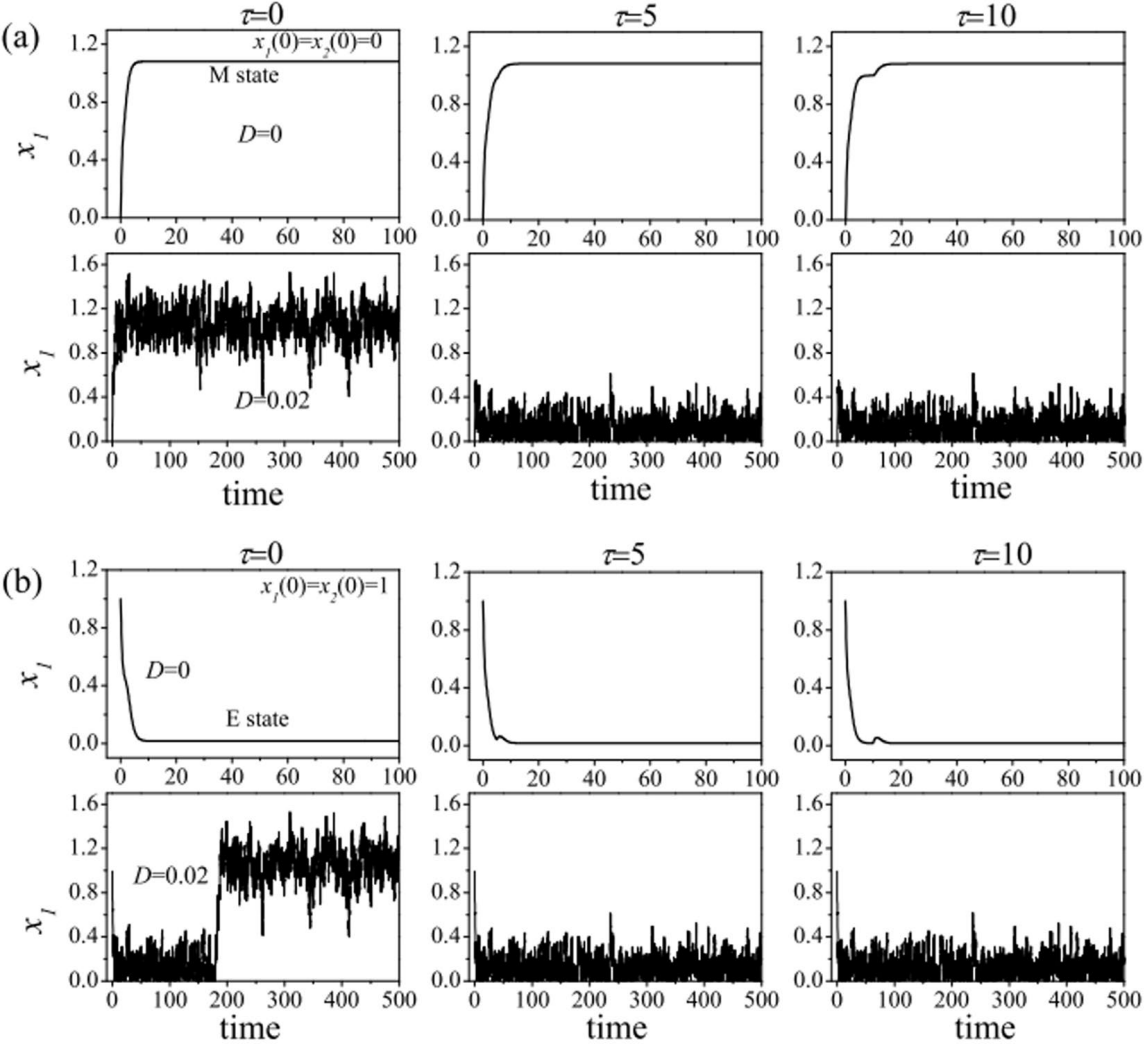
Effects of time delay and noise on the expression of ZEB at the point (*a* = 0.1, *b* = 1) in a bistable region of phase diagram. (**a**) The initial state of cancer cell is in the E state, (**b**) The initial state of cancer cell is in the M state.

In the absence of noise, when the cancer cell starts in the M state or in the E state (i.e., the steady state of expression level of *x*_1_ over the time, not the initial value of *x*_1_). With the increasing of time delays, Fig. 8(a,b) (the top) show that the expression level *x*_1_ of ZEB is independent of the time delay *τ*, only depends on the initial state of cancer cell.

In the presence of noise, however, Fig. 8(a,b) (the bottom) show that the expression level *x*_1_ of ZEB is not only depend on the time delay but also on the initial state of cancer cell. When the cancer cell starts in the M state (Fig. 8(a)), the cancer cell transforms from M to E state (i.e., M → E) with the increasing of time delay. When the cancer cell starts in the E state (Fig. 8(b)), with the increasing of time delay, the cancer cell firstly transforms from E to M state, and then to E state (E → M → E).

#### In the tristable region

**Figure 9:**
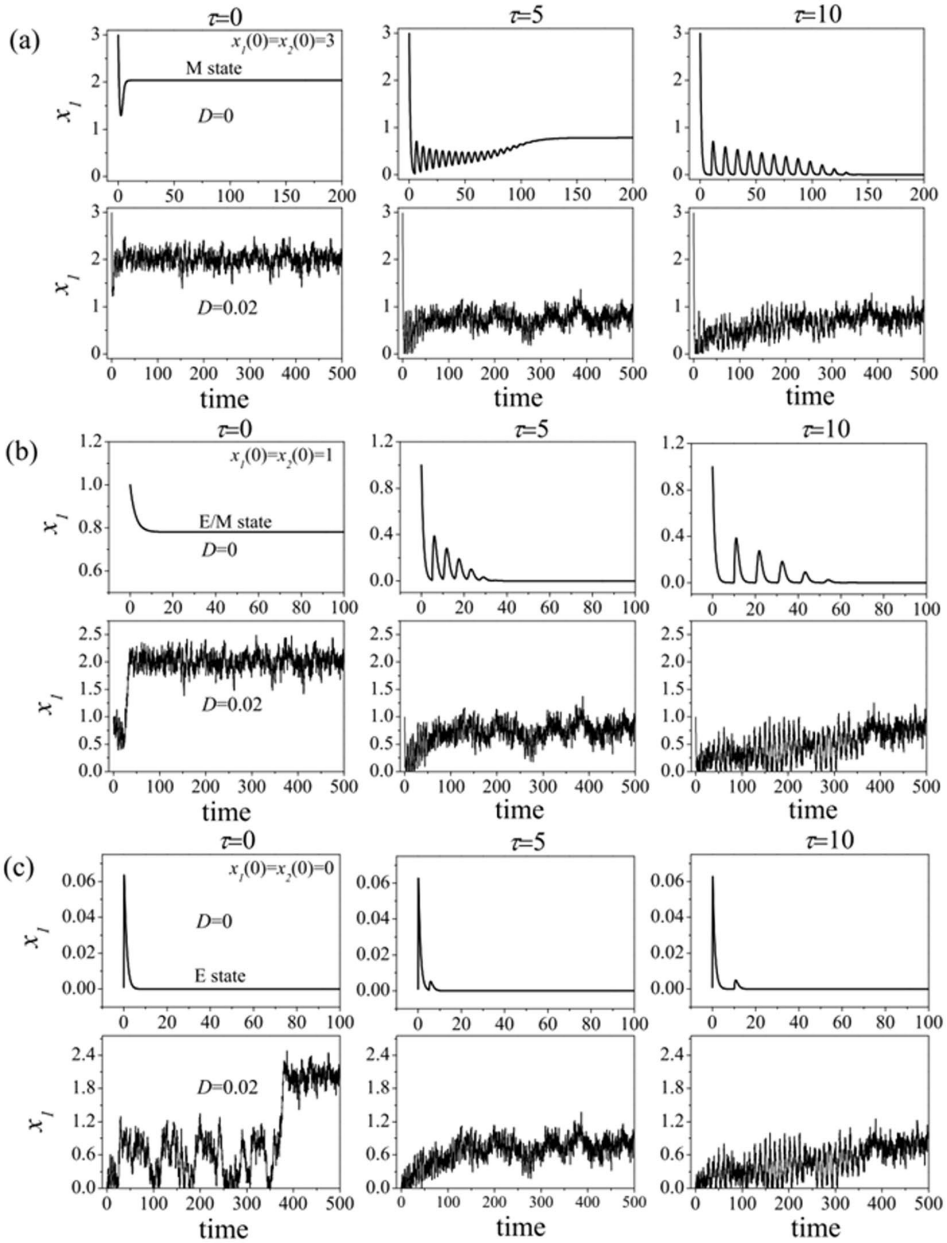
Effects of time delay and noise on the expression of ZEB at the point (*a* = 1.1, *b* = 8) in tristable region of phase diagram. (**a**) The initial state of cancer cell is in the E state, (**b**) The initial state of cancer cell is in the E/M hybrid state. (**c**) The initial state of cancer cell is in the M state.

In the absence of noise, when the cancer cell starts in the M state, Fig. 9(a) (the top) shows that, with the increasing of time delay, the expression level *x*_1_ is firstly oscillation, and then reaches to a middle constant level, and finally to low constant level. It implies that the cancer cell undergoes a succession of transitions: from M to E/M hybrid, and then to E state (M → E/M → E). When the cancer cell starts in the E/M hybrid state, Fig. 9(b) (the top) shows that, with the increasing of time delay, the expression level *x*_1_ is firstly oscillation, and then reaches to a low constant level. It implies that the cancer cell undergoes a transition from E/M hybrid to E state (E/M → E). When the cancer cell starts in the E state, Fig. 9(c) (the top) shows that, with the increasing of time delay, the expression level *x*_1_ is firstly oscillation, and then reaches to a low constant level, it implies that the cancer cell stays in the E state at all time.

In the presence of noise, with the increasing of time delay, when the cancer cell starts in the M state, Fig. 9(a) (the bottom) shows that the cancer cell undergoes a transition from M to E/M hybrid (M → E/M). When the cancer cell starts in the E/M hybrid state, Fig. 9(b) (the bottom) shows that the cancer cell undergoes a succession of transitions: from E/M hybrid to M state, and then from M to E/M hybrid state (E/M → M → E/M). When the cancer cell starts in the E state, Fig. 9(c) (the bottom) shows that the cancer cell may undergo a succession of transitions: from E to E/M hybrid state, and from E/M hybrid to M state, and then to E/M hybrid state (E → E/M → M → E/M).

Above results show that the effects of noise on the expression level *x*_1_ of ZEB are opposite to these of time delay. Noises can induce low expression level of ZEB switching to high expression level ZEB (i.e., E → E/M or E/M → M), however, time delay can induce high expression level of ZEB switching to low expression level ZEB (i.e., E/M → E or M → E/M). Therefore, there is competition between noise and time delay in the phenotypic states transitions process when they are considered simultaneously.

## Conclusions and Discussions

In this paper, based on the core regulatory network proposed by Lu *et al*.^19^, a general minimal dynamic model with two variables is proposed to understand the EMT and MET processes and the effects of both noise and time delay on phenotypic transitions of cancer cell. By utilizing the stability analysis of dynamic model, it is shown that single cancer cell exits three phenotypic states: the epithelial state, the mesenchymal state, and the epithelial hybrid mesenchymal state. The phase diagram of dynamic model shows that there are monostable, bistable, and tristable states regions in a parameters plane, that is, the cancer cell exists the coexistence of multiple phenotypic states, and interconverts between different states.

By using the bifurcation analysis and the stationary probability of genes expression level, it is found that different pathways in the phase diagram correspond to the EMT or the MET process of cancer cells, and there are two EMT paths. The phenomenon of E/M hybrid state in the EMT processes but rather in the MET process observed by experiments^11,12^ can be understood through different pathways in the (*a*, *b*) parameters plane. In the multiple states (E, E/M, M) coexistence region, it is found that the transitions in phenotypes of cancer cells can be induced by noise. With the increasing of noise intensity, the mean first passage time and the height of barriers of two attractors show that the cancer cell has a large probability of transition into the M state through the E/M hybrid phenotype. It is found that the barrier height of E/M hybrid phenotype is firstly decreased, and then reaches a platform value when the noise intensity is larger than *D*_*c*_ = 0.03, that is, when *D* > *D*_*c*_, the E/M hybrid state always exists no matter how large the noise intensity is. The cancer cell in the hybrid state can acquire the capabilities of resistance and robustness to against the high pressure environment. It was reported that cancer cells in the E/M hybrid phenotype exhibit stemness characters during EMT in developmental, regenerative, as well as pathological contexts^48–51^, and related to drug resistance^52–54^.

The numerical simulations show that the effects of noise on multiple phenotypic states transitions are opposite to these of time delay, and there is competitive mechanism between noise and time delay in the multiple phenotypic transitions process. In conclusion, a general minimal dynamic model with two variables is proposed to understand the EMT and MET processes and the effects of both noise and time delay, and the phenomenon of E/M hybrid state in the EMT process but rather in the reverse MET process observed by experiments is revealed through different pathways in a parameters plane (phase diagram).

Epithelial to mesenchymal transition (EMT) plays an important role in embryonic development, tissue regeneration, and cancer metastasis. By using our minimal model, it was found that there are three states of each cancer cell, the states could be reproduced by using the model of miR-200/ZEB module. The three states are (high miR-200/low ZEB) or (1, 0), (low miR-200/high ZEB) or (0, 1), and (medium miR-200/medium ZEB) or (1/2, 1/2), which correspond to the epithelial phenotype (E), mesenchymal phenotype (M) and epithelial/mesenchymal hybrid (E/M), respectively.

Compared our results with previous work^19^, the phases and bifurcation diagrams looked differences, the reasons for the differences are (i) the parameters in our general minimal model are dimensionless, and the parameters in the model of core regulatory network^19^ are dimension. (ii) The assumptions of genes switching functions (or Hill functions) in both models are different. However, the biological significance of phases and bifurcation diagrams are the same. For example, by using our minimal dynamic model, the phase diagram of phenotypic states of single cancer cell can be drawn in parameters (*a*, *b*) plane as shown by Fig. 2, the phase diagram can reproduce the coexistence of multiple phenotypic states and the phenotypes transitions of cancer cells: two monostable regions which correspond to the E sate or the M state, two bistable regions which correspond to the coexistence of E and M states or the coexistence of E and E/M hybrid states, and a tristable region which corresponds to the coexistence of E, E/M hybrid, and M states. Importantly, by using our general minimal model, it is found that the E state always exist until the the activation strength a of ZEB is too large in the bifurcation diagrams (see Figs 4(a)–6(a)). Our results suggest that the E state may be a fundamental phenotype for the cancer cell which is consistent with the experimental observations^46,47^.

Our general minimal model is involved in negative regulation between microRNA and transcription factor such as miR-200/ZEB. It is well known that RNA interference and microRNA are new technologies in drug development. For example, Olmeda *et al*.^55^ disclosed that specific silencers of endogenous miRNAs, antagomirs, are powerful tools to silence specific miRNAs *in vivo*. Iorio and Croce^56^ reviewed the involvement of microRNAs in cancer and their potential as diagnostic, prognostic, and therapeutic tools. Wang *et al*.^57^ discussed the CSCs, EMT and the role of regulation by miRNAs in the context of drug resistance, tumor recurrence and metastasis. Therefore, microRNAs associated with EMT such as miR-200 family could be exploited as therapeutic strategies in the future. Therefore, our general minimal model for the miR-200/ZEB module could provide new insights into the roles of microscopic regulatory mechanisms, noise, and time delay in single cancer cell, and the dynamic model might give some insights for various tumors clinical therapy strategies.
